# Somebody to Lean on: Understanding Self-Stigma and Willingness to Disclose in the Context of Addiction

**DOI:** 10.3390/ijerph21081044

**Published:** 2024-08-08

**Authors:** Qing Ma, Christopher R. Whipple, Övgü Kaynak, Erica Saylor, Weston S. Kensinger

**Affiliations:** School of Behavioral Sciences and Education, Penn State University, Harrisburg, Middletown, PA 17057, USA; qpm5012@psu.edu (Q.M.); crw5586@psu.edu (C.R.W.); ems6002@psu.edu (E.S.); wsk120@psu.edu (W.S.K.)

**Keywords:** opioid use disorder, self-stigma, mutual support group, disclosure

## Abstract

Substance use self-stigma is a barrier to treatment and can negatively impact individuals’ well-being and treatment engagement. Given the mixed findings in previous research and the limited specific investigation into the concept of self-stigma within the context of opioid misuse, examining factors associated with self-stigma in the context of opioid use disorder (OUD) is warranted. The current study examines the influence of individual-level factors (race, sex, urban/rural status, support group attendance) on self-stigma and willingness to disclose opioid use. Data for this study were from a larger study of OUD-related stigma among adults in Pennsylvania, U.S. The current study included participants who indicated a personal past or current history with OUD were included (*n* = 84). Exploratory factor analysis and multiple indicators, multiple causes (MIMIC) model were used to explore the associations between demographic factors (i.e., sex, age, race/ethnicity, urban/rural status), attendance at mutual support groups, and self-stigma factors. Results indicated that sex and attendance at mutual support groups significantly predicted levels of self-stigma. Women and individuals with no previous experience attending mutual support groups endorsed lower levels of self-stigma. Additionally, attendance at mutual support groups predicted willingness to self-disclose past and present opioid use. Individuals who reported no history of attending mutual support groups demonstrated less willingness to disclose past and present OUD use compared to participants who were support group attendees. The current research findings enhance the understanding of OUD-related self-stigma by examining its relationship with individual-level factors, disclosure, and attendance to mutual support groups. The results offer insights into the influence of sex and support group attendance on self-stigma and disclosure. These findings have significant clinical implications for developing future interventions and promoting health policy changes.

## 1. Introduction

The opioid overdose epidemic continues to be a public health crisis with a reported 79,770 opioid-related overdose deaths taking place in the year 2022 [1]. The opioid crisis in the United States was triggered in the late 1990s by a significant increase in the prescription and consumption of opioid pain medication, a consequence of marketing campaigns launched by pharmaceutical companies [2,3]. The heightened exposure to prescription opioids resulted in a greater risk of addiction and overdose. Unfortunately, of the 54.6 million individuals (aged 12 and above) that needed substance use disorder treatment in 2022, only 4.6 percent of these Americans (13 million) received substance use treatment [4]. Less than 1% of individuals with an opioid use disorder (OUD) received medication-assisted treatment (MAT) for opioid use in 2022 [4]. Additionally, only 2% of individuals reported attending a support group and less than 1% of individuals reported receiving service from peer support specialists or recovery coaches [4]. Substance use stigma plays a part in answering the question of why individuals with a substance use disorder decide not to pursue substance use treatment—in one study, the majority (78.2%) of respondents with substance use disorders reported that they should be able to handle their alcohol or drug use on their own and almost half (46.1%) worried what people might say if they got treatment [4].

The stigma associated with addiction has been investigated as a complex concept encompassing negative stereotypes that persist in the broader structural environment, including social and cultural contexts [5,6]. Erving Goffman defined stigma as a disparaging attribute that minimizes an individual’s social identity, leading to negative stereotypes, discrimination, and devaluation by others. Research on stigma reveals unique stereotypes, prejudice, and discrimination associated with people with addiction [7,8]. Feldman and Crandall pointed out that stigmatized social rejection and distance were more likely when individuals were perceived as personally responsible for their psychological conditions and considered dangerous or threatening to others within the same environment, especially with rare conditions like substance use disorder [9].

### 1.1. Public Stigma and OUD

Prior research comparing the public stigmatization associated with mental illness and addiction-related illnesses suggests that people tend to perceive individuals with addiction-related diagnoses as more blameworthy for their conditions and more dangerous to the public compared to mental illness-related diagnoses, implying more overall stigmatization toward individuals with substance use diagnoses [10]. Individuals who struggle with substance use report feeling socially alienated and demonstrate tendencies to conceal their addiction from their family, friends, and coworkers to avoid feeling discriminated against or judged [11]. Individuals with OUD, in particular, are frequently perceived by the public as having criminal tendencies, subpar work performance, and a diminished moral compass [12]. Public stigma is associated with various negative outcomes that threaten individuals’ physical and emotional safety. Latkin and colleagues reported that addiction-related discrimination from others increased overdose risk and was associated with recent and lifetime overdose history [13]. Stigma towards individuals with substance use disorder is associated with low engagement in social support and treatment seeking, adverse emotional states, poor mental health outcomes, and difficulties in aspects such as employment, housing, and socialization [5,13,14,15,16]. Furthermore, experiences and perceptions of stigma within a social and cultural context can be internalized by individuals with OUD [5,16].

### 1.2. Self-Stigma and OUD

Self-stigma, or internalized stigma, refers to the process by which individuals with OUD accept and internalize the negative stereotypes and judgments from their environment attached to their condition [6,17,18]. Modified labeling theory posits that individuals tend to internalize negative public perceptions and external stigmatizations associated with their identities [19]. It is possible that people form their identities as their self-concept develops based on the social groups to which they perceive themselves to belong [20]. Self-stigma can negatively impact self-perception, increase psychological distress, and decrease overall quality of life [21]. When individuals with OUD perceive that they are judged, ostracized, discriminated against, or mistreated by others due to their disorder, they also experience negative emotional states such as feeling shame/guilt, fear, hopelessness, low self-worth, poor self-esteem, negative self-perceptions, and diminished self-value [15,17,22,23].

The adverse psychological consequences of self-stigma can manifest as negative behavioral outcomes, such as withdrawal or avoidance, which can impede individuals’ willingness to seek help, disclose their condition, engage in treatment, or utilize available support systems [11,15,16,24]. The negative impact on self-disclosure could lead to increases in self-stigma perpetuating the cycle of avoiding help-seeking behaviors.

### 1.3. Individual-Level Factors Associated with Self-Stigma

Previous studies examining the individual-level factors (e.g., sex, age, race, rural/urban status) associated with substance use self-stigma have been limited. What research is available is inconsistent, emphasizing the necessity for further investigation. For example, when considering sex and self-stigma, previous studies have produced mixed results. While some studies indicated that men reported higher levels of self-stigma [25,26,27], others suggested that women exhibited more self-stigma [24,28], or found no significant differences between men and women at all [29,30].

Research examining age and substance use self-stigma has also been sparse. One prior study examined the relationship between individuals’ attitudes toward mental health service utilization and associated self-stigma [31]. Results indicated that older participants (65 years or older) reported the lowest level of self-stigma and perceived public stigma related to help-seeking behaviors compared to younger (18–29) and middle-aged (40–64) participant groups (18 to 39 years old) [31]. Conversely, another study found a small positive correlation between age and how much the individual internalized derogatory stereotypes [25]. Overall, much of the research examining both sex and age in relation to self-stigma has been limited to mental health literature [32,33,34].

Browne and colleagues pointed out that one’s racial background can compound with substance use stigma and form additional treatment-seeking barriers [35]. A limited number of studies examined OUD-related self-stigma with a focus on participants’ racial backgrounds. Preceding addiction-related studies on individuals’ racial backgrounds and self-stigma yielded mixed results based on individuals’ racial and cultural identities. A study conducted within the U.S. military population revealed that White men reported higher mental health self-stigma scores compared to their Black/African American peers [30]. In a separate study, Rivera and colleagues found that Latino participants reported higher self-stigma related to substance use compared to the White/Caucasian participants [36].

The distinction between rural and urban status may also play a role in influencing substance use self-stigma and mental health concerns. However, few quantitative studies exist and findings have been mixed. One study that examined differences in substance use self-stigma between rural and urban individuals found no significant difference in the levels of self-stigma based on individuals’ rural and urban status [25]. Another study investigated addiction treatment barriers and motivations among pregnant women and found that, compared to rural women, urban women reported stigma as more of a barrier to treatment [37]. To our knowledge, no research articles have focused on individuals’ rural and urban status when examining OUD-related self-stigma.

Finally, mutual support groups are a form of supportive intervention for individuals in OUD recovery that may also impact self-stigma. Attending Narcotics Anonymous (NA) meetings is an effective recovery support that allows self-determination of attendees’ involvement in the treatment process, provides social support, and is free [38]. According to NA World Services, NA holds more than 72,000 meetings weekly in 143 countries worldwide [39]. Tracy and Wallace reviewed ten studies on the effects of mutual support groups in substance use treatment and found that groups were helpful in assisting attendees with their recovery in terms of post-discharge sobriety, relapse reduction, and community engagement [40]. Reif and colleagues supported the positive impact of mutual support groups and found that support group attendance was associated with individuals’ relapse reduction, increased treatment retention, better interpersonal relationships, and more satisfaction with the treatment experience [41]. Attendance at mutual support groups can help individuals who use opioids maintain their sobriety with participants attending weekly mutual support groups demonstrating long-lasting positive effects on abstinence [42]. In addition to improving self-efficacy, attending mutual support groups is also associated with improvements in perceptions of stigmatizing beliefs of others [43] and self-stigma [44]. One prior research found that participation in mutual support groups and decreased self-stigma significantly affected the quality of life reported by participants with severe mental health concerns [45]. While research has demonstrated the positive effects of mutual support groups, research on how mutual support group attendance impacts OUD self-stigma and disclosure is sparse.

### 1.4. Current Study

Given the mixed findings in previous research and the limited specific investigation into the concept of perceived self-stigma within the OUD population, it is crucial to examine related research areas to enhance understanding of the effects and factors associated with self-stigmatization in the context of OUD recovery. To explore demographic factors for self-stigmatization and contribute to the existing literature on related subjects, this study investigates the associations between perceptions of self-stigma, demographic factors, and treatment-seeking behaviors concerning OUD disclosure and treatment. We have hypothesized that demographic factors, including sex, age, race/ethnicity, urban/rural status, and participation in mutual support groups, are predictive indicators of self-stigmatization among individuals with OUD. More specially, we hypothesized that rural Caucasian younger males would report higher levels of self-stigma compared to other demographic groups. Additionally, we hypothesized that individuals who had attended mutual support groups would report lower levels of self-stigma compared to those who had not attended mutual support groups.

## 2. Materials and Methods

### 2.1. Participants

Data for this study were from a larger study of OUD-related stigma among adults in Pennsylvania, U.S. (*N* = 1033). For the current study, only those participants who indicated a personal past or current history with OUD were included (*n* = 84). The majority of participants were male (53.6%), non-Hispanic (95.2%), and White (95.2%), with an average age of 42.2 years (*SD* = 12.7 years). A full demographic breakdown may be seen in Table 1.

### 2.2. Research Design

Full details of the design of the larger study may be found in Kaynak et al., 2022 [46]. Adults were recruited across Pennsylvania to participate in a cross-sectional survey of OUD-related stigma. Participants were recruited by a market research company from existing pools of web survey panels. The overall study utilized quota-based sampling to produce a sample representative of adults in Pennsylvania by age, sex, and region. Data collection occurred between June and July 2020. For the current study, only participants with a past or current OUD were utilized; as such, the sample for the current study is not representative of Pennsylvanian adults.

### 2.3. Measures

The survey instrument for the overall study included 73 items, measuring public, workplace, policy, and self-stigma. When the original project began in 2020, there was no comprehensive validated addiction-focused stigma survey instrument. Thus, the study team, consisting of addiction experts from a number of areas (e.g., research, non-profit, academic, clinical) identified items from a number of related surveys measuring stigma toward SUD or other related constructs (e.g., mental health) and developed study-specific items, where necessary (see [46]). Some items were adapted to fit the current focus on OUD; others were used as is. For the current study, 13 study-specific self-stigma items were derived from existing mental health self-stigma measures [47]. All items were measured on a 5-point Likert scale (1 = Disagree strongly to 5 = Agree strongly). Four items measured participants’ comfort level sharing their OUD history with others (e.g., “I feel comfortable talking to my employer about my present or past opioid use”, “I feel comfortable talking to my friends about my present or past opioid use”, “I feel comfortable talking to my family about my present or past opioid use”, “I feel comfortable talking to my doctor about my present or past opioid use”). Four items measured what participants believed others would feel about them if they knew about the participant’s OUD (e.g., “People think I am worthless if they know about my opioid use history”, “If someone were to find out about my history of opioid use, they would doubt my character”, “People around me will always suspect I have returned to using opioids”, “People will think I have little talent or skill if they know about my opioid use history”). Five items measured activities participants may have avoided or concerns participants may have had about disclosing their OUD history to others (e.g., “I have avoided applying for a job because I worried that someone would stigmatize me because of my opioid use”, “I am worried that people could find out about my present or past opioid use”, “I would be afraid to seek help for a relapse because it implies that I have no willpower”, “I have avoided meeting new people because of my opioid use”, “I would avoid treatment because I don’t want people to find out about my present or past opioid use”). In addition to stigma questions, participants were asked about specific demographic characteristics, including their sex (1 = male, 2 = female), age, race/ethnicity (1 = White, non-Hispanic, 2 = Not White, non-Hispanic) (as defined by the U.S. Census Bureau) [48], and urban/rural status (1 = rural, 2 = urban). Lastly, participants were asked about their attendance at mutual support groups (“Have you ever attended any self-help group or 12-step program for your opioid use?”; 1 = yes, 2 = no).

### 2.4. Procedures

Research activities were approved by The Pennsylvania State University Institutional Review Board. Participants were recruited from existing web survey panels and were identified using existing participant profiles. Participants eligible to participate were contacted and provided study information and a unique link to complete the survey on Qualtrics. Participants first answered screening questions to validate eligibility and to determine quota needs in the quota-based sampling system. A total of 1033 participants completed the survey. For the current study, only the participants who indicated a history of OUD were selected (*n* = 84).

### 2.5. Analytic Strategy

To identify factors associated with perceived self-stigma among individuals with a history of OUD, multiple analyses were completed. First, exploratory factor analysis (EFA) was used to reduce the self-stigma items into latent factors of self-stigma. To accomplish this, principal axis factoring was completed in SPSS version 29. To determine how many factors to extract, a scree plot was examined, and a parallel analysis was performed. After factor extraction, an oblique rotation (direct oblimin) was used to interpret the factors. After interpreting the results of the EFA, factors were included in a multiple indicators, multiple causes (MIMIC) model. MIMIC models are specialized structural equation models in which multiple observed indicators are used to estimate latent factors and latent factors are regressed on multiple predictor variables [49]. First, we specified a measurement model using confirmatory factor analysis (CFA), using the EFA factors previously determined. Model fit was assessed using several indicators, including the chi-square test of model fit, the root mean square error of approximation (RMSEA) [50], comparative fit index (CFI) [51], and standardized root mean square residual (SRMR) [52]. Good-fitting models were indicated by a non-significant chi-square value, RMSEA values below. 06, SRMR values below. 08, and CFI values close to or greater than. 95 [50,53,54]. After examining model fit for the CFA, a MIMIC model was analyzed to examine associations between demographic factors (i.e., sex, age, race/ethnicity, urban/rural status), attendance at mutual support groups, and self-stigma factors. Despite the sample size, we employed factor analysis due to the high factor loadings (factor 1 had 5-factor loadings greater than 0.6, and factor 2 had 4-factor loadings greater than 0.6), how to factor number, and relatively large number of variables, which suggested a reliable structure [54,55,56].

## 3. Results

The Kaiser–Meyer–Olkin (KMO) measure of sampling adequacy (KMO = 0.81) indicated adequate data to conduct factor analysis. Bartlett’s test of sphericity, χ^2^(78) = 476.04, *p* < 0.001, indicated significant correlations between indicator variables. To determine the number of factors to extract, a scree plot and a parallel analysis were examined. Observation of the scree plot indicated that two factors were to be extracted. The separate parallel analysis results supported the factor extractions indicated by the scree plot. After extracting two factors, an oblique rotation (direct oblimin) was conducted, and the pattern matrix was evaluated to interpret factors. The first factor had nine items with high factor loadings (e.g., “People think I am worthless if they know about my opioid use history”, “If someone were to find out about my history of opioid use, they would doubt my character”). We labeled this factor “perceived self-stigma”. The second factor had four items with high factor loadings (e.g., “I feel comfortable talking to my friends about my present or past opioid use”, “I feel comfortable talking to my doctor about my present or past opioid use”). We labeled this factor “willingness to disclose”. The two factors were not strongly correlated with each other (*r* = −0.29).

Prior to testing the MIMIC model, the measurement model of self-stigma and willingness to disclose was tested using Confirmatory Factor Analysis. Confirmatory Factor Analysis indicated adequate model fit (χ^2^(63) = 95.04, *p* = 0.006; RMSEA = 0.08; CFI = 0.93; SRMR = 0.07). Although the chi-square test of model fit was significant, other indicators suggested adequate fit. After fitting the measurement model, indicators were added as predictors of the latent factors in a MIMIC model. The MIMIC model had an adequate fit (χ^2^(118) = 151.75, *p* = 0.020; RMSEA = 0.06; CFI = 0.93; SRMR = 0.07). Participants’ sex (*β* = −0.53, *p* = 0.013) and attendance at mutual support groups (*β* = −0.54, *p* = 0.023) were significant predictors of perceived self-stigma. Specifically, women and individuals who had not attended a mutual support group reported significantly less perceived self-stigma than men and individuals who had attended a mutual support group. Other predictors were not significantly associated with perceived self-stigma. Only attendance at mutual support groups significantly predicted willingness to disclose (*β* = −0.57, *p* = 0.011). Specifically, attendance at mutual support groups was associated with a greater willingness to disclose. Please see Table 2 for test statistics for all predictors in the MIMIC model. The results of the current study indicated that participants’ racial and geographical backgrounds did not generate significant outcomes. However, our hypothesis of sex as a significant predictor of self-stigmatization was supported by our results. Additionally, our hypothesis that attendance at mutual support groups would be associated with less self-stigma was partially supported; attendance at mutual support groups was associated with a higher willingness to disclose one’s OUD status, but more perceived self-stigma.

## 4. Discussion

The current study examined the relation between participants’ individual-level factors, including demographics and mutual support group attendance with OUD self-stigma and disclosure. Factor analysis was utilized due to the reliable structure characterized by high factor loadings, low factor numbers, and a high number of variables [54,55]. First, results suggested that sex and attendance at mutual support groups significantly predicted levels of self-stigma. More specifically, women and individuals with no previous experience attending mutual support groups endorsed lower levels of self-stigma. Second, findings indicated that attendance at mutual support groups predicted willingness to disclose past and present opioid use. Particularly, individuals who reported no history of attending mutual support groups demonstrated less willingness to disclose past and present OUD use compared to participants who were support group attendees.

Consistent with existing studies on sex-related risk factors, results revealed a sex disparity in OUD self-stigma. In this sample, women reported less perceived self-stigma compared to men which supports some prior research in this area [24,28]. There are potential sex differences in processing shame, a common emotion experienced by individuals seeking treatment, which could potentially help explain the sex differences in self-stigma found here. As individuals recognize and begin to come to terms with their substance use and current situation, personal shame can emerge [57]. Women tend to link shame with guilt while men associate shame with embarrassment [30]. It is possible that increased efforts to promote the medical model of addiction which focuses on OUD as a brain disease and not as a personal failing, may make it easier to process and let go of feelings of guilt. However, this may not ease feelings of embarrassment as individuals reflect on past behaviors associated with their substance use. Finding ways to help men, in particular, process shame surrounding their past substance use may be warranted.

The connection between a lack of prior engagement in mutual support groups and a reduced perception of self-stigma stresses the varying role that support groups could play in stigmatization. Self-stigma is common among individuals in substance use treatment [29]. Prior findings indicate that higher levels of self-stigma are associated with longer stays in residential treatment, implying a lowered sense of self-efficacy and more fear of being stigmatized by those outside a more protected treatment setting [58]. Thus, it is possible that individuals with lower levels of self-stigma may feel capable of asking family members or friends for support or seeking treatment in other settings, like primary care or local hospital settings. Further, mutual support groups are often used in tandem with drug specialty treatment and can improve outcomes including treatment engagement and sobriety [59,60]. It is possible that those who utilize mutual support groups may have more acute or severe substance use disorders warranting more engagement in the drug treatment system. Unfortunately, individuals who seek help for their substance use within the healthcare system can experience stigma from the people who are supposed to be there to help them [61,62]. Hospital-based providers and individuals with lived experience can attest to the stigma experienced within the healthcare system, from in-person interactions to documentation in medical charts [63]. Thus, the individuals who reported attending mutual support groups in this sample may have been more actively engaged in treatment, potentially increasing their exposure to stigmatization through disclosing their status and interacting with medical providers, fellow support group attendees, and people in their social and environmental settings, an experience less common for those not in treatment or support groups.

Furthermore, support group attendees may experience heightened self-stigma as they become more educated on addiction-related concepts and are increasingly more aware of various negative impacts of their OUD that were previously unrecognized. In other words, individuals attending support groups could be at a more advanced stage of recovery with more self-awareness of stigma compared to non-attendees. According to SAMHSA and the Center for Substance Abuse Treatment [64], people who attend mutual support groups are at the stage where they recognize the necessity for taking action to initiate change and attain a more advanced understanding of how their substance use adversely affects their own lives and the lives of others. This dynamic could inadvertently contribute to stronger negative emotions surrounding their substance use disorder at the beginning of their treatment process, which could be the potential explanation for the increased self-stigma. Though this negative phase may seem counterproductive initially, it is worth noting that this phenomenon might project a subsequent stage of growth in one’s journey of recovery, where things might worsen temporarily before improving. Erikson’s psychosocial development theory supports this growth trajectory that people grow and advance into the next stage of development after resolving their developmental crises or challenges at a given stage, which could cause initial stress and discomfort but eventually lead to growth and stage advancement [65].

Consistent with previous research on the benefits of attending mutual support groups, results suggested that individuals who did not participate in support groups were less inclined to disclose their OUD history. Prior research demonstrated the positive outcomes of attendance to support groups, including reduced substance use and relapse prevention [40,41,66]. Similarly, the current results further confirm the benefits of attending mutual support groups with a particular emphasis on the stigma-reducing effects peer support groups have on attendees. It could be that mutual support groups foster an environment with comfort and openness for attendees to disclose their OUD history, which could help reduce the barriers to disclosure.

## 5. Conclusions

### 5.1. Limitations

Our research possesses certain limitations. Due to the characteristics of the analyses conducted for the current research, no casual conclusion could be drawn. The cross-sectional self-reporting format of the data collection yields its limitations, such as varied degrees of personal insights and response bias. Additionally, participants volunteering in the current study entailed a non-probability sample which limited representation of the population. The overall sample size is relatively small for these types of analyses. Smaller sample sizes may be associated with a number of issues, including inflated standard errors and inflated Type I error rates [67]. Future research should replicate this study with larger sample sizes of people with OUD and incorporate objective data-collecting methods to mitigate the limitations of self-reporting. Moreover, future research could integrate qualitative data regarding clients’ specific experiences and perceptions of the impact of peer support groups. Additionally, the current research findings provide clinical implications for understanding self-stigma within the context of sex and substance use treatment. As suggested by our results, OUD-related self-stigma may be different based on sex and temporarily influenced by treatment attendance. Furthermore, the self-stigma measure utilized in this study was specifically designed for the research in this paper, with items derived through factor analysis. Although self-stigma and perceived social stigma are two distinct constructs, there may be concept overlap between them, complicating the measurement of self-stigma in isolation from perceived social stigma. Future research should focus on developing addiction-specific self-stigma measures to assess this concept more comprehensively. Lastly, while the model fit for our CFA and MIMIC models was adequate based on the criteria described in the Analytic Strategy, the model fit may be in doubt if applied to more strict criteria [68]. Despite limitations, the current study provides much-needed insight into self-stigma among individuals with OUD.

### 5.2. Future Directions

When developing interventions for OUD treatment, it is crucial to consider potential sex differences that may impact treatment outcomes. This is especially important for treatments focused on reducing self-stigma and for developing stigma-related psychoeducation materials. Sex-specific stigma-reduction interventions and support groups should be tailored to address the unique needs of different sexes with OUD. These interventions should take into account individuals’ substance misuse patterns and sex, employing a comprehensive approach that considers intersectionality. For instance, self-stigma and associated clinical barriers that could potentially prevent men from seeking treatment or attending support groups should be considered when designing interventions or promoting support groups.

Previous research documented the prevalence of significant stigmatizing attitudes among healthcare providers toward individuals with substance use disorders [60,61]. More specifically, a recent study revealed that primary care physicians exhibit notably stigmatizing attitudes toward individuals with OUD [69]. Considering healthcare professionals tend to experience severe OUD cases in their work, such as emergency room admissions for overdose, medication-seeking behaviors, or severe injection-related injuries, skewed or potentially biased perceptions and views may be formed in this process that impact the larger healthcare environment and consequently affect patients’ treatment experience and their willingness to disclose past or present substance use. More OUD stigma reduction-focused training could be implemented in various clinical settings for healthcare providers and staff to gain educational awareness and reduce substance misuse-related myths and biases. In addition, it would be beneficial to integrate more comprehensive services into the general healthcare system, particularly within primary care practices given that many individuals with OUD do not require intensive 30-day inpatient programs. Therefore, incorporating addiction services into routine primary care could help healthcare professionals develop a more balanced and less stigmatized view of individuals with OUD. Reducing social stigma towards patients seeking help for their substance use may impact levels of self-stigma and break the cycle of public and internalized stigma, and, in turn, improve levels of disclosure and treatment-seeking behavior.

## Figures and Tables

**Table 1:** Participant Demographic Characteristics.

Variable		*n*	%
Sex	Male	45	53.6
	Female	39	46.4
Race	White	80	95.2
	Black	5	6
	Asian	1	1.2
	American Indian or Alaska Native	2	2.4
	Something else	1	1.2
Ethnicity	Non-Hispanic	80	95.2
	Hispanic	4	4.8
Residence	Rural	25	29.8
	Urban	59	70.2

Note. Age of the participants was *M* = 42.2, *SD* = 12.7.

**Table 2:** Predictor and Outcome Variables.

	Self-Stigma & Perceived Judgement			Willingness to Disclose		
	*β*	*t*	*p*	*β*	*t*	*p*
Sex *	−0.53	−2.49	0.013 *	−0.06	−0.27	0.784
Age	−0.16	−1.16	0.248	0.17	1.57	0.117
Race	0.40	1.28	0.201	0.09	0.29	0.774
Rural/Urban Status	−0.10	−0.41	0.680	0.15	0.51	0.613
Support Group Attendance *	−0.54	−2.28	0.023 *	−0.57	−2.53	0.011 *

* Indicates significant results.

## Data Availability

The data used in this study are publicly available and can be accessed at https://padashboard.lifeunitesus.com/ or by contacting the corresponding author.
